# Exploring Hormone Therapy Effects on Reproduction and Health in Transgender Individuals

**DOI:** 10.3390/medicina59122094

**Published:** 2023-11-29

**Authors:** Efthalia Moustakli, Orestis Tsonis

**Affiliations:** 1 Laboratory of Medical Genetics, Faculty of Medicine, School of Health Sciences, University of Ioannina, 45110 Ioannina, Greece; thaleia.moustakli@gmail.com; 2 Fertility Preservation Service, Assisted Conception Unit, Guy’s and St Thomas’ NHS Foundation Trust, London SE1 9RT, UK

**Keywords:** transgender patients, gender-affirming hormonal treatment, transgender health, reproductive health, mental well-being, fertility preservation

## Abstract

Transgender individuals often face elevated mental health challenges due to gender dysphoria, but gender-affirming treatments such as surgery and hormone therapy have been linked to significant improvements in mental well-being. The potential influence of time and circadian rhythms on these treatments is prevalent. The intricate interplay between hormones, clock genes, and fertility is profound, acknowledging the complexity of reproductive health in transgender individuals. Furthermore, risks associated with gender-affirming hormonal therapy and potential complications of puberty suppression emphasize the importance of ongoing surveillance for these patients and the need of fertility preservation and family-building options for transgender individuals. This narrative review delves into the intricate landscape of hormone therapy for transgender individuals, shedding light on its impact on bone, cardiovascular, and overall health. It explores how hormone therapy affects bone maintenance and cardiovascular risk factors, outlining the complex interplay of testosterone and estrogen. It also underscores the necessity for further research, especially regarding the long-term effects of transgender hormones. This project emphasizes the critical role of healthcare providers, particularly obstetricians and gynecologists, in providing affirming care, calling for comprehensive understanding and integration of transgender treatments. This review will contribute to a better understanding of the impact of hormone therapy on reproductive health and overall well-being in transgender individuals. It will provide valuable insights for healthcare providers, policymakers, and transgender individuals themselves, informing decision-making regarding hormone therapy and fertility preservation options. Additionally, by identifying research gaps, this review will guide future studies to address the evolving healthcare needs of transgender individuals. This project represents a critical step toward addressing the complex healthcare needs of this population. By synthesizing existing knowledge and highlighting areas for further investigation, this review aims to improve the quality of care and support provided to transgender individuals, ultimately enhancing their reproductive health and overall well-being.

## 1. Introduction

Individuals who identify as transgender or nonbinary (TNB) often grapple with significantly elevated rates of mental health challenges, including feelings of sadness, anxiety, and even thoughts of suicide and suicide attempts as a result of gender dysphoria (GD) that these patients experience [1]. However, there is compelling evidence to suggest that gender-affirming surgery (GAS), gender-affirming hormonal treatment (GAHT), and puberty blockers (PBs) can independently contribute to mitigating these adverse mental health outcomes [2]. In particular, research has illuminated the transformative potential of these interventions. They have been associated with reduced levels of anxiety, depression, and other negative mental health experiences among TNB individuals [3]. Moreover, for adults who had the opportunity to access PBs during adolescence, these interventions have been linked to a decreased lifetime prevalence of suicidal ideation.

While the positive influence of gender-affirming care on mental well-being is increasingly evident, the precise impact of such care on immediate mental health outcomes following initiation remains a topic of ongoing exploration. Several studies published between 2015 and 2020 have highlighted the positive effects of receiving PBs and/or GAHT [4]. These effects encompass enhanced psychological functioning, improved body satisfaction, diminished sadness, and reduced tendencies toward suicidality, all observed within the span of a year [5]. Furthermore, it is worth noting that the initial stages of receiving gender-affirming care coincide with clinical staff’s affirmation of an individual’s gender identity, potentially contributing to short-term improvements in mental health. Intriguingly, the circadian clock, which governs the rhythmic orchestration of physiological and behavioral activities within the human body in response to environmental cues, plays a vital role in this narrative. The harmonious synchronization between internal circadian rhythms and external environmental cycles is crucial for maintaining optimal health. Disruptions in this synchronization can lead to various pathological conditions [6].

Hence, a groundbreaking therapeutic approach known as “chronotherapy” has emerged, focusing on leveraging the concept of time to optimize disease treatment [7,8]. This approach recognizes that, beyond regulating sleep and endocrine functions, circadian rhythms profoundly influence reproductive rhythms. Recent scientific investigations have unveiled the intricate connections between circadian clock genes and the intricate processes governing hormone release in key areas such as the hypothalamus, pituitary gland, ovaries, and various other endocrine organs [9]. In essence, the hypothalamic–pituitary–gonadal (HPG) axis, which governs the entire reproductive system, operates within a precisely timed framework [9,10]. Any disruption to these circadian rhythms can potentially trigger a range of pathological disorders and diseases. This revelation underscores the intricate interplay between circadian biology and reproductive health, shedding new light on potential avenues for therapeutic intervention [11,12]. This narrative review aims to investigate hormone therapy’s impact on transgender individuals’ fertility and general health in a comprehensive manner. Furthermore, this review offers a comprehensive understanding of the ways in which hormone therapy affects transgender populations’ reproductive results and other health indicators by investigating the physiological and psychological effects of this intervention. The objective of this study is to provide significant insights that can improve treatment plans, guide healthcare practices, and improve the health of transgender patients receiving hormone therapy.

## 2. Materials and Methods

For this review, four major search machines were explored, i.e., MEDLINE, Google Scholar, PubMed, and EMBASE, using the following keywords and possible combinations of them: transgender; gender-affirming hormonal therapy; GAHT; gender-affirming surgery; GAS; mental health; gender dysphoria; circadian rhythm; complications; fertility; fertility preservation. Data were collected regarding mental health and gender dysphoria, time and circadian rhythms, hormones, clock genes, risks and complications, bone and cardiovascular health, as well as interventions including GAHT, puberty suppression, and fertility preservation, into a conceptual or theoretical framework. Due to the lack of sufficient evidence, this review summarizes the current knowledge based on systematic reviews, as well as retrospective and prospective studies on transgender patients, focusing only on patients receiving GAHT and on their fertility. Exclusion criteria were scientific evidence addressing mainly moral implications or attitudes towards family building in the transgender community.

## 3. Circadian Rhythm, Time, and How Do Hormones and Clock Genes Affect Fertility?

Chronotherapy, an emerging field in medical science, investigates the optimal timing of medical interventions to enhance treatment efficacy and minimize side effects. Its foundational premise lies in recognizing the body’s inherent circadian rhythms, the biological cycles that regulate physiological processes over a 24 h period [13,14]. While the concept has gained notable traction in various medical disciplines, its application in the realm of gender-affirming treatments is currently limited by a lack of robust data. The potential influence of circadian rhythms on hormonal fluctuations, particularly those associated with gender identity and expression, suggests that the timing of interventions may play a crucial role in optimizing treatment outcomes. In the context of gender-affirming treatments, the intricate dance between circadian rhythms and reproductive hormones becomes particularly relevant. While examining the temporal aspects of these processes provides a compelling avenue for exploration, it is important to acknowledge that, at present, there is limited empirical support for the specific implementation of chronotherapy in the optimization of gender-affirming treatments [15].

In addition to circadian rhythm and the impact of time, the intricate molecular machinery governed by clock genes was initially discovered in the cells of diverse tissues across various species. Subsequently, researchers unveiled a compelling connection between these clock mechanisms and fertility as well as reproductive success. This relationship, it turns out, operates in a bidirectional manner, as reproductive hormones also exert an influence on clock-gene expression, creating a complex web of interactions. Fertility, a finely orchestrated process, is under the meticulous regulation of the hypothalamic–pituitary–gonadal axis (HPG axis), accompanied by two crucial neuronal populations within the hypothalamus: Kisspeptin neurons and gonadotropin-releasing hormone (GnRH) neurons [10]. These components work in concert to ensure reproductive success. The anterior ventral periventricular Kisspeptin neurons, for instance, play a pivotal role in orchestrating the luteinizing hormone (LH) surge, a critical event in the reproductive cycle [16]. Meanwhile, the hypothalamic Kisspeptin neuron population, located within the arcuate nucleus, serves a dual purpose. It not only imparts crucial metabolic status information to the HPG axis but also releases GnRH, further propelling the intricate dance of reproductive hormones [17,18].

The intricate dance continues as the hypothalamus releases gonadotropin-releasing hormone (GnRH) in pulsatile fashion. GnRH, in turn, acts upon the anterior pituitary lobe (adenohypophysis), instigating the synthesis of gonadotropins—follicle-stimulating hormone (FSH) and LH—before finally releasing them into the bloodstream. In males, LH plays a pivotal role in testosterone production, while FSH contributes to the generation of sperm. On the female front, FSH and LH collaborate with ovarian follicles to produce essential steroid hormones, specifically estrogens. Notably, the peak of LH triggers continuous expression of the BMAL1 gene and its corresponding protein in the mouse ovary, underscoring the role of circadian rhythms in reproductive processes. However, any dysfunction in FSH signaling can lead to male subfertility and impaired spermatogenesis [19]. Similarly, alterations in Kisspeptin signaling can result in hypogonadotropic hypogonadism, affecting Leydig cell development, germ cell advancement, and sperm functionality. In essence, this intricate interplay between hormones and clock genes underscores the profound complexity of the mechanisms governing fertility, offering a deeper understanding of the delicate balance required for successful reproduction.

## 4. The Role of Sex Hormones

Estrogens encompass a group of compounds capable of binding to one or both human estrogen receptors, ER1 and ER2, thereby eliciting a response. In the human body, endogenous estrogens are primarily produced by the placenta and ovaries [20,21,22]. These endogenous human estrogens are 18-carbon steroids with varying binding affinities and potency. In addition to natural human estrogens, numerous organic and synthetic substances, both steroidal and nonsteroidal, can bind to estrogen receptors, acting as agonists and/or antagonists [23]. These compounds fall under the category of selective estrogen receptor modulators (SERMs), with natural human estrogens constituting a special subclass [24]. Because estrogens are hydrophobic steroids and do not readily enter the intestine or dissolve in the blood, they rely on carrier proteins to facilitate their transport from their source to target tissues [25]. This characteristic is of paramount importance when selecting estrogens for prescription and determining the method of administration. For instance, 17β-estradiol exhibits more efficient absorption at certain interfaces compared to the gastrointestinal tract, allowing for lower prescribing doses [26]. Additionally, 17β-estradiol can permeate mucous membranes and skin more rapidly than the intestinal mucosa. To enhance intestinal absorption and prolong the half-life, estrogens can be conjugated with other molecules [27]. Estrogens efficiently interact with cell membranes. Upon reaching target cells, they bind to cytoplasmic estrogen receptors and subsequently translocate from the cytoplasm to the nucleus, where they exert a wide array of effects [23].

Estrogen receptors are found in most human tissues, mediating a broad spectrum of physiological effects induced by estrogens and further reinforcing that the link between the circadian system and estrogen production is the discovery of ER receptor expression in the suprachiasmatic nucleus (SCN) [23,25,27,28]. The onset of puberty marks the commencement of heightened estrogen production in the ovaries, leading to accelerated growth of the skeleton, particularly the long bones. This period is also characterized by the accumulation of subcutaneous fat tissue, notably in the hip and thigh areas, as well as the initiation of mammary gland development, resulting in breast formation. Furthermore, the maturation of the Mullerian system is signaled by the development of endometrial growth and bleeding. The cyclic growth of follicles and ovulation necessitates both positive and negative feedback of estrogen to the central nervous system, facilitating the maturation of the hypothalamic–pituitary–ovarian axis. These pubertal transformations are accompanied by substantial maturation and psychosocial development, typically stabilizing over a span of 3 to 5 years. Long-term exposure to estrogen appears to influence the quantity and distribution of visceral and subcutaneous fat and may contribute to the decelerated growth of intra-arterial plaques. While evidence on the long-term preservation of cognitive function is mixed, estrogens may help maintain neuronal dendritic density. Estrogens also inhibit bone resorption, thus reducing the risk of osteoporosis and fractures [25,26].

In addition to estrogens, androgens also exert a significant influence on fertility. Androgen levels follow a diurnal cycle, peaking in the morning [29]. Androgens play indispensable roles in the development and maintenance of secondary sexual characteristics, libido, growth, osteoporosis prevention, and spermatogenesis in males. In females, LH governs the synthesis of ovarian androgens [28]. The elevated production of androgens, a common characteristic of female hyperandrogenemia, notably observed in polycystic ovary syndrome (PCOS), has been shown to impact the expression of the Clock gene in rat ovarian follicles [30]. PCOS patients frequently experience obesity and metabolic syndrome, conditions that are closely associated with reduced fertility, irregular menstrual cycles, and diminished gonadotropin output. Additionally, androgens exert direct, tissue-specific effects on Per2 gene expression, potentially elucidating their role in shaping the developmental program of the timing system.

## 5. Risks Associated with GAHT

The primary sources informing our understanding of the risks associated with gender-affirming hormonal therapy (GAHT) are the well-documented dangers associated with menopausal hormone therapy and combined hormonal contraception [31]. However, it is important to note that extrapolating these risks to transwomen is challenging due to variations in dosage, concomitant hormones, and treatment duration among this population [32]. Moreover, there is a paucity of specific data pertaining to transwomen [33]. It is crucial to recognize that for many transwomen seeking sex reassignment hormone therapy, it is not merely a choice but a necessary treatment to align their physical characteristics with their gender identity [34].

One of the most immediate and concerning risks of estrogen therapy is the well-established increase in venous thromboembolic events (VTE) [35]. These events encompass conditions such as myocardial infarction, stroke, pulmonary embolism, and cardiovascular mortality. Notably, the WHI estrogen-progestin study reported a twofold increase in the overall risk of VTE, albeit with a modest absolute increase in event incidence (0.18%) [32]. In contrast, an estrogen-only trial showed a 33% increase in VTE risk, resulting in an actual event incidence rise of 0.07%. The route of estrogen administration is a significant factor contributing to thromboembolic hazards [36]. When taken orally, estradiol (E2) and conjugated equine estrogen (CEE) undergo a “first-pass” effect in the liver, leading to elevated prothrombotic factors [36]. However, evidence from the Million Women’s Study suggests that non-oral administration, primarily through transdermal patches, does not carry an increased risk of VTE compared to a control population [37]. The safety profile of sublingual and injectable non-oral delivery methods is less well documented, but they are likely to align more closely with transdermal or transvaginal delivery rather than oral preparations [38].

Research on VTE risk in transwomen receiving estrogen remains limited. An analysis of electronic medical records from 2842 transwomen at 3 Kaiser Permanente sites revealed that transwomen had a 16.7- and 13.7-fold higher risk of VTE after 2 years of therapy compared to same-sex men and women, respectively. However, these results were not mirrored for acute myocardial infarction. Notably, the majority of transwomen in this study were taking oral estradiol. Ethinyl estradiol (EE), another form of estrogen, was associated with a significant increase in VTE risk in one study, with the risk decreasing upon discontinuation of EE. A broader international evaluation reported a VTE rate of less than 1%. In addition to VTE, other short-term risks encompass hypertriglyceridemia, the potential induction of prolactinoma growth, hypertension, and cholelithiasis [39]. Extremely limited information is available regarding the long-term effects of estrogen therapy in transwomen, primarily because comprehensive long-term studies are lacking. A study examining the cumulative experiences of 2236 transwomen and 876 transmen over 32 years found no increase in mortality, including cardiovascular mortality [36,39,40]. While few hormone-related malignancies were identified, the use of oral ethinyl estradiol was discontinued due to a significantly elevated risk of VTE. No comparable increase in risk was noted with other estrogen preparations. Of particular concern in the long term is the elevated risk of breast cancer in transwomen. Presently, there are insufficient statistical data to quantify this risk accurately [36].

## 6. The Role of Gender-Affirming Hormonal Therapy (GAHT)

**Figure 1:**
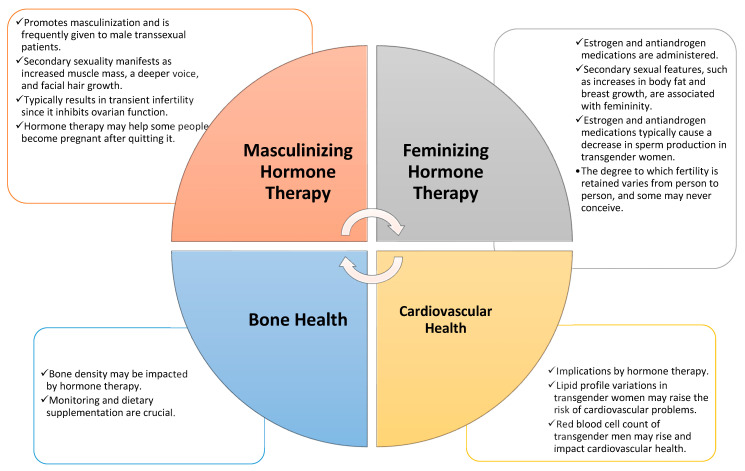
GAHT and health considerations.

Gender-affirming hormone therapies (GAHT) for transwomen typically incorporate additional medications that either inhibit circulating androgens, primarily testosterone, or directly reduce androgen synthesis (T) [41]. Lowering serum testosterone levels to fall within the normal female range is crucial for halting terminal hair growth in areas containing androgen receptors. These areas, especially in sexually dimorphic regions such as the chin, cheeks, sternum, upper abdomen, and upper back, are highly sensitive to even slight elevations above this range, which can stimulate further hair growth [42,43]. In cases where estrogen doses, adequate for feminization, prove insufficient for complete androgen suppression, the use of an antiandrogen becomes imperative for favorable clinical outcomes [44,45]. Antiandrogens can be categorized into three groups: those that interfere with androgen-receptor signaling, those that limit the conversion of testosterone to its potent metabolite, dihydrotestosterone (DHT), and those that reduce androgen production [46]. Although substantial clinical data exist regarding their relative efficacy and adverse effects, practical considerations such as cost and administration methods often dictate the choice of antiandrogen in a given treatment regimen [47] (Figure 1).

The initiation of testosterone therapy brings about several expected changes within three months, including amenorrhea (absence of menstruation), increased facial and body hair, skin alterations, acne flare-ups, alterations in fat distribution and muscle mass, and heightened libido [48]. Subsequent side effects include voice deepening, vaginal epithelial atrophy, clitoral enlargement, and, over time, male-pattern hair loss due to androgenic interaction with pilosebaceous units [5,49]. It is essential to note that while some individuals perceive these changes as masculinizing, others may find them beneficial. For those experiencing hair loss and not finding it desirable, 5-Reductase inhibitors can be considered as an adjunct treatment [3,4]. Patients should be educated about the potential impact of these drugs on sexual function, alongside the fact that there is limited information regarding their use in transgender men. Except when testosterone is administered during the peri-pubertal period, most female-to-male patients retain some degree of feminization, which cannot be reversed by exogenous testosterone. Consequently, many transgender men typically have smaller physiques, exhibit a more feminine subcutaneous fat distribution, and often possess wider hips compared to cisgender men [50].

Upon commencing estrogen therapy, the following changes typically occur: breast enlargement, increased body fat, reduced growth of body and facial hair, testicular size reduction, and improved erectile function. The extent of these changes varies among individuals and may take 18 to 24 months to fully manifest [22]. Combining antiandrogenic therapy with other treatments optimizes these outcomes. Hormone therapy enhances the quality of life for transgender individuals and has been associated with positive effects on mood and sexual function in longitudinal studies. These biological findings align with research on serotonin reuptake transporter binding (SERT) in trans men and women [51]. Patients with major depressive disorder often exhibit lower SERT expression, and studies have shown that estrogen and antiandrogen medications reduce regional SERT binding in transwomen, whereas androgen therapy increases SERT binding in different brain regions in transmen. These preliminary data emphasize the therapeutic significance of hormone treatment in addressing gender dysphoria and potentially alleviating physiological stress. For instance, a study by Colizzi et al. found that taking cross-gender hormones significantly reduced cortisol levels and subjective stress in transgender patients after 12 months of treatment [52,53].

The impact of GAHT on cognitive domains remains uncertain, particularly in terms of improvements relevant to daily functioning [54]. While GAHT generally aligns transgender individuals’ cognitive features with their identified gender, the practical applicability of this information to the majority of trans individuals and their healthcare providers for day-to-day quality of life may be limited. Future research should shift focus away from sex-specific cognitive processes and instead explore brain regions associated with goal-directed behavior [55,56]. Over the past few decades, studies have indicated that transgender individuals report reduced anxiety, perceived stress, and social distress following GAHT, accompanied by improvements in mental health-related quality of life, self-esteem, and mood. Nevertheless, certain disparities regarding the effects of GAHT on anger and aggression require further investigation [57].

The prefrontal cortex, responsible for regulating executive function and influenced by estrogen, plays a pivotal role in cognitive activities such as sustained attention, working memory, organizing, and planning [58]. Transgender men (FTM) receiving high doses of testosterone may experience executive dysfunction, similar to what is observed in cisgender females with premature estradiol loss or those with polycystic ovary syndrome [3]. Given that many individuals anticipate lifelong GAHT, the field requires additional high-quality longitudinal research to assess a diverse range of behavioral and cognitive domains over time, with and without neuroimaging. Moreover, future studies should address healthcare disparities throughout the entire gender spectrum and expand their scope to include genders beyond the binary [59].

## 7. Complications of Puberty Suppression

Puberty suppression for premature puberty is generally a safe medical intervention with few acute and long-term side effects. Adverse reactions primarily manifest at the injection site, with up to 9% of patients experiencing redness and swelling and 10% to 20% reporting local pain. While the initial dose of GnRH agonists stimulates gonadotropins and temporarily increases pubertal changes, these effects are usually transient [60]. Patients may experience emotional lability, mood swings, testicular pain, acne exacerbation, and, in female Tanner-4 individuals, even vaginal bleeding at the onset of therapy [61]. Suppression of the hypothalamic–pituitary–gonadal (HPG) axis results in hypogonadism symptoms among older adolescents who have already initiated puberty with puberty blockers [62]. These symptoms may include fewer erections, increased shaving frequency, vaginal discomfort, itching, hot flashes, fatigue, and subjective weakness. Another expected consequence of suppressing puberty is changes in body composition, typically involving decreased lean body mass and increased body fat percentage, effects that appear to persist only until the onset of hormone therapy [63]. While transgender hormone therapy affects fertility, puberty suppression itself has minimal impact on fertility outcomes in transsexuals [64].

Recent research has raised concerns about the potential adverse effects on bone health, likely linked to increased use of GnRH agonists [63,65]. Initially, there is apprehension that bone mineral accumulation could be compromised due to the absence of sex hormones during puberty suppression. However, these concerns seem to subside over time once sex hormones (either endogenous or exogenous) are reintroduced. In transgender adolescents who undergo longer periods of puberty suppression, there is heightened anxiety about potential skeletal issues [66,67]. Studies in patients with precocious puberty have yielded conflicting findings regarding the impact of GnRH on bone mineral density (BMD) [68,69]. Nevertheless, emerging evidence suggests that any adverse effects on BMD may reverse upon discontinuation of GnRH treatment and the resumption of puberty. While limited, studies involving transsexual participants have shown that BMD increase is delayed during GnRH treatment. This effect persisted even after six years of cross-sex steroid use in both sexes. However, it is important to note that all participants in these studies were in the late pubertal or post-pubertal stage. In a recent study, transsexual adolescents treated with GnRH agonists exhibited lower markers of bone turnover and apparent bone mineral density. This effect was successfully reversed after two years of cross-sex steroid treatment [70].

## 8. Impact of GAHT on Reproduction and the Role of Fertility Preservation

Histopathological studies have shown that the use of testosterone in GAHT may result in polycystic-like ovaries and variable effects on the endometrium, including atrophy and proliferation. However, findings from these studies are not consistent [71,72]. Spontaneous pregnancies have been reported after discontinuation of testosterone, but more research is needed to determine the long-term effects of testosterone on ovarian and endometrial function as well as fertility [73]. The timing of fertility preservation for transgender individuals is critical and should be based on various factors, including the individual’s age, gender identity, medical history, and GAHT plans. Fertility preservation is ideally offered after puberty and before any medical or surgical gender-affirming interventions. Postponing such interventions until after fertility preservation may exacerbate feelings of distress and discomfort associated with gender dysphoria [74]. However, it is important to consider that oocyte quality is known to decline with age, and peak female fertility occurs in the early 20s. Therefore, the timing of fertility preservation should take into account the patient’s age and individual circumstances, as well as the impact of GAHT [75]. Additionally, fertility experts should communicate the age-related decline in oocyte quantity and quality, particularly to transgender males seeking fertility preservation. Patient discussions about these topics should occur before starting gender-affirming treatment to ensure informed decision-making.

For transgender males opting for controlled ovarian stimulation (COS), the duration of the procedure should be anticipated, and potential stressors should be discussed in advance. Patients should be advised about the use of Mirena coils to minimize the risk of bleeding during the process, and transabdominal ultrasound should be considered for follicular tracking. When discontinuing GAHT is acceptable, the addition of letrozole to reduce circulating estrogen during COS could be considered, but more research is needed to establish the psychological benefits of these interventions [76,77]. In the case of transgender females, sperm cryopreservation is most effectively achieved through ejaculated sperm obtained through masturbation. Patients should be informed that even if they decide to preserve testicular tissue during gender-affirming surgery, spermatogenesis cannot currently be achieved clinically. Adequate counseling should be offered to patients who may be reluctant to discontinue GAHT. Clinicians must be aware of the potential mental and emotional implications of GAHT cessation on gender dysphoria and offer alternative options [78,79].

Transgender individuals have various options for family building, including pregnancy, surrogacy, and gamete donation. Adoption is often preferred, as it does not require GAHT discontinuation [80]. Adequate information about the legal aspects and limitations of these options should be provided to patients. A multidisciplinary approach, involving fertility experts, endocrinologists, and psychologists with expertise in transgender care, is crucial to address the unique challenges faced by transgender individuals. By adopting a more concise and organized approach to presenting the information, this revised text provides a clearer and more reader-friendly account of the complexities and considerations involved in fertility preservation for transgender individuals [81].

## 9. Surveillance for Transgender Population

In both males and females, the sex steroids testosterone and estradiol are required for the maintenance of bone health. Both men and women suffering from a hypogonadal condition may experience clinically significant bone loss as they monitor bone formation and turnover [61]. Although testosterone has a direct function in maintaining bone health, it is also peripherally aromatized with estradiol, which plays a critical role. Further aiding in the maintenance of bone health is the significant role of testosterone in increasing muscle mass. Bone health studies have been conducted in transgender men receiving long-term testosterone therapy [82]. When exogenous testosterone is administered in physiologic amounts, it appears to have an anabolic effect on cortical bone and is sufficient to prevent bone demineralization problems in transgender patients. In addition, when transgender women take estrogens, they may be more susceptible to bone loss. Because antiandrogen use likely is to blame, health professionals should consider discontinuing antiandrogen therapy when patients receive orchiectomy, regardless of whether genital confirmation surgery is also performed [69].

It is unclear whether the use of exogenous testosterone makes transgender men more susceptible to cardiovascular disease. According to certain research, testosterone adversely affects variables that could increase the risk for cardiovascular events [83]. For example, available evidence demonstrated that long-term use of testosterone leads to an increase in triglycerides and inflammatory indicators, whereas high-density lipoprotein cholesterol decreases. A study on 50 patients who had taken testosterone for an average of 10 years found that many of the patients had high blood pressure and high serum lipids [84]. Despite these metabolic changes and their adverse effects on putative cardiovascular disease risk factors, no study found an increase in the incidence of cardiovascular events such as myocardial infarction, deep vein thrombosis, and cerebrovascular events.

Although research on transgender women and the effects of estrogen on cardiovascular disease is not very conclusive, it suggests that there may be a trend toward increased risk of heart disease that needs further investigation [85]. Patients should refrain from taking oral ethinyl estradiol as their main treatment because it appears to be highly associated with cardiovascular events. In addition, diabetes has been associated with a higher risk of cardiovascular morbidity in transwomen taking estrogen, as this comorbidity is common in the transgender community. Diabetes is a major risk factor for cardiovascular disease. There is a lack of comprehensive prospective studies in the literature. Few of the individuals studied are older than 65 years, and many of the currently available studies have limited patient numbers and short- or medium-term follow-ups [85,86,87]. In addition, no studies have been published comparing hormone preparations side by side. Therefore, it is impossible to draw firm conclusions about the adverse effects of long-term use of transgender hormones [87].

Comprehensive and supportive healthcare for transgender patients necessitates a collaborative, multidisciplinary approach involving various stakeholders. The primary care physician serves as the initial point of contact for medical care, playing a crucial role in assessing overall health, delivering gender-affirming care, and providing essential medical services [88,89]. Additionally, endocrinologists focus on the hormonal aspects of transgender health, utilizing hormone replacement therapy (HRT) to induce desired secondary sexual traits aligned with an individual’s gender identity. While some transgender individuals opt for surgery to align physical attributes with their gender identity, specialized surgeons in transgender healthcare perform procedures such as genital reconstruction (vaginoplasty, phalloplasty), face feminization, and breast augmentation or reduction [90]. Pediatricians are instrumental in providing necessary care for transgender children and adolescents, offering support, monitoring growth, and addressing interventions such as puberty blockers or teenage hormone therapy when needed. Mental health specialists, including psychologists and therapists, contribute significantly to transgender healthcare by addressing concerns such as depression, anxiety, and overcoming social obstacles. They also play a crucial role in assessing a patient’s readiness for gender-affirming therapy. Andrologists, specialists in male reproductive health, may contribute to transgender healthcare, particularly for individuals transitioning from an assigned female at birth (AFAB) to a male identity, offering services related to sexual health, fertility preservation, and reproductive health [91].

The importance of a multidisciplinary team (MDT) in transgender care cannot be overstated. An MDT ensures seamless coordination among healthcare professionals, optimizing the delivery of comprehensive services. The collaborative nature of an MDT allows for a holistic understanding of the patient’s needs, facilitating a more tailored and personalized approach to care. Communication and information sharing among team members become paramount, fostering a more cohesive and patient-centered experience. Additionally, an MDT provides a platform for ongoing education and training, ensuring that healthcare professionals stay informed about the evolving landscape of transgender healthcare. The collective expertise of diverse specialists within the team contributes to a more nuanced understanding of the complexities involved in transgender care, ultimately enhancing the quality of services and promoting the mental, emotional, and physical well-being of transgender individuals [92].

## 10. Future Considerations in Transgender Care

General obstetricians and gynecologists are well qualified to provide hormone therapy for transgender patients, affirming their gender identity. However, further research is essential to enhance our capacity to address reproductive issues and prescribe relevant medications [60]. It is of utmost importance that primary care providers, including gynecologists, become well versed in these treatments and integrate them into their practice. This demand arises as more individuals seek gender-confirming hormone therapy, necessitating vigilant monitoring and the involvement of knowledgeable healthcare providers [8].

Laboratory monitoring of transgender patients on hormone therapy can be challenging due to gender-specific reference ranges that may not be suitable for this population. Few published data exist on reference ranges for important cardiovascular and metabolic parameters, apart from cholesterol, triglycerides, hemoglobin, and hematocrit. Roberts et al. [93] conducted a study on male–female patients undergoing hormone therapy and found that certain parameters such as low-density lipoprotein, hemoglobin, and hematocrit aligned with typical female values. However, levels of potassium, creatinine, and alkaline phosphatase resembled those of men. Most notably, triglyceride levels were higher than the reference values for both men and women. This study underscores the need to establish specific reference ranges for transgender women to prevent diagnostic errors.

While this analysis focuses on best practices and patient management for adults undergoing hormone therapy, it is essential to consider special considerations for adolescents with gender dysphoria. Typically, cross-sex hormones are recommended from around age sixteen. However, in some cases, earlier therapy initiation may be necessary to prevent psychological and cognitive distress in adolescents [1]. A multidisciplinary approach and parental support are crucial in adolescent care. GnRH agonists are used to suppress endogenous hormones in adolescents at Tanner stage 2 to prevent full pubertal development. Cross-sex hormone therapy can begin at age 16 or earlier. Ethical challenges should be addressed, making it advisable to entrust the treatment of adolescent transgender patients to experienced medical professionals [2].

Mepham et al. [94] performed a cross-sectional study that revealed that a significant portion of transwomen self-prescribe cross-gender hormones, often through the Internet. Another study found that nearly half of transwomen in San Francisco were taking hormones not prescribed by a physician [56]. While the likelihood of obtaining hormones from unofficial sources is decreasing as more physicians gain experience in hormone therapy, patients should be screened for out-of-home use and informed of associated risks. Patients might perceive obstacles if they are not provided hormones immediately or are instructed to seek psychiatric treatment before starting hormone therapy. It is crucial for patients to understand that physicians are not excluding them from therapy but are striving to ensure a successful outcome. This reiterates the importance of a multidisciplinary approach to treatment for transgender patients.

**Figure 2:**
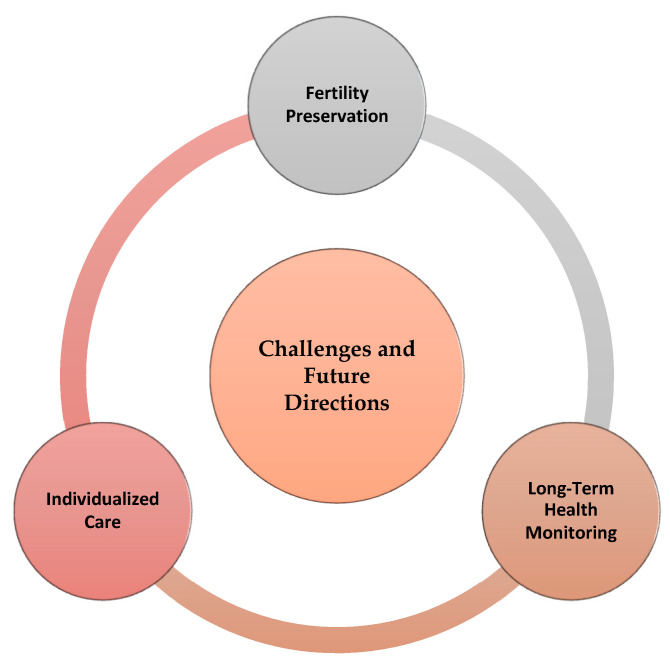
Challenges and future directions.

**Figure 3:**
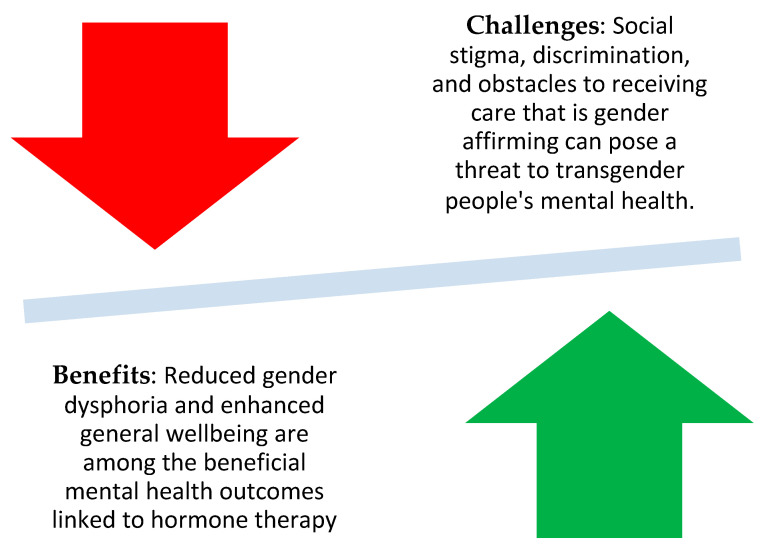
Mental health considerations.

While the objectives of gender-affirming hormone therapy for adult the trans population are conceptually clear, challenges persist in their practical implementation. These individuals have undergone pubertal development, resulting in an adult body that does not align with their gender identity. The primary aim is to reverse male sex development as extensively as possible and simultaneously advance trans sex development without incurring unacceptable long-term risks [95]. This dual objective encompasses genetic and hormonal changes, affecting traits such as reproductive organ function and body shape. Ensuring the long-term safety of lifelong medication is equally imperative. It is essential to conduct comprehensive risk assessments and establish strategies for risk mitigation. However, it is crucial to recognize that, for many transgender people, gender reassignment hormone therapy is not a luxury but a necessity to align their bodies with their identity. The occasional tension between the perceived need for therapy and potential risks necessitates appropriate support from medical professionals [96] (Figure 2 and Figure 3).

In conclusion, the multifaceted landscape of transgender healthcare encompasses a spectrum of considerations, spanning from the impact of gender-affirming hormone therapy (GAHT) on reproduction to the role of fertility preservation and the broader implications on cardiovascular and metabolic health. The critical importance of a multidisciplinary team (MDT) in transgender care cannot be overstated, as it ensures a coordinated and comprehensive approach, optimizing the delivery of services and addressing the unique challenges faced by transgender individuals. The timing of fertility preservation emerges as a crucial factor, balancing the individual’s age, gender identity, and GAHT plans. Future considerations in transgender care emphasize the need for continued research to enhance our understanding of reproductive issues and medication management, with a call for primary care providers to integrate gender-affirming hormone therapy into their practice. Challenges in laboratory monitoring, special considerations for adolescents, and the importance of a multidisciplinary approach underscore the evolving nature of transgender healthcare. As the field progresses, ongoing education, specialized care coordination, and a nuanced understanding of the complex interplay between medical, psychological, and social factors remain paramount for the holistic well-being of transgender individuals.

## Data Availability

Not applicable.
